# Supplementary material for: Cultivar-specific nutritional status of potato (Solanum tuberosum L.) crops
Source: PLoS One. 2020 Mar 13;15(3):e0230458. doi: 10.1371/journal.pone.0230458 (PMC7069643; doi:10.1371/journal.pone.0230458)
Supplement: S2 Table — (DOCX) [file pone.0230458.s002.docx]

**S2 Table: Description of potato data set used for cluster analysis**

| **Cultivar** | **Maturity class** | **Number obs.** | | **Yield cut-off** | **Median clr values** | | | | | |
| --- | --- | --- | --- | --- | --- | --- | --- | --- | --- | --- |
|  |  | **High yielders** | **Total** | **Mg ha^-1^** | **N** | **P** | **K** | **Ca** | **Mg** | **Fv** |
| **AC Belmont** | early | 21 | 60 | 29.87 | 0.70 | -2.01 | 0.66 | -1.11 | -1.62 | 3.46 |
| **AC Chaleur** | early | 6 | 23 | 16.70 | 0.70 | -1.97 | 0.35 | -1.04 | -1.54 | 3.51 |
| **Amandine** | mid-season | 2 | 6 | 35.42 | 0.52 | -2.29 | 0.56 | -0.70 | -1.62 | 3.53 |
| **Ambra** | mid-season | 2 | 6 | 53.69 | 0.71 | -1.98 | 0.28 | -1.08 | -1.39 | 3.46 |
| **Andover** | early mid-season | 11 | 30 | 39.97 | 0.87 | -2.17 | 0.74 | -0.81 | -2.28 | 3.57 |
| **Aquilon** | mid-season | 23 | 66 | 24.60 | 0.82 | -1.86 | 0.28 | -0.91 | -1.83 | 3.48 |
| **Argos** | late | 7 | 20 | 45.07 | 0.77 | -1.77 | 0.57 | -1.28 | -1.70 | 3.43 |
| **Atlantic** | mid-season | 88 | 184 | 36.82 | 0.78 | -1.89 | 0.17 | -1.10 | -1.50 | 3.57 |
| **Bijou Rouge** | early | 9 | 24 | 36.53 | 0.94 | -1.93 | 0.56 | -1.12 | -1.93 | 3.61 |
| **Carolina** | early | 3 | 8 | 39.03 | 0.60 | -2.19 | 0.13 | -0.84 | -1.34 | 3.63 |
| **Chieftain** | mid-season | 119 | 258 | 36.89 | 0.77 | -1.92 | 0.40 | -1.06 | -1.91 | 3.60 |
| **Coastal Russet** | mid-season | 58 | 165 | 33.12 | 0.67 | -1.86 | 0.39 | -0.95 | -1.84 | 3.51 |
| **Dark Red Chieftain** | mid-season | 8 | 22 | 36.81 | 0.96 | -1.71 | 0.36 | -1.37 | -1.86 | 3.65 |
| **Estima** | mid-season | 12 | 35 | 54.85 | 0.93 | -2.13 | 0.58 | -0.98 | -1.83 | 3.53 |
| **FL 1207** | mid-season late | 127 | 346 | 36.33 | 0.74 | -2.09 | 0.28 | -0.93 | -1.51 | 3.45 |
| **FL 1533** | mid-season | 64 | 183 | 36.20 | 0.63 | -1.80 | 0.22 | -1.13 | -1.35 | 3.47 |
| **Frontier Russet** | mid-season | 9 | 24 | 34.28 | 0.52 | -2.20 | 0.62 | -0.37 | -2.06 | 3.58 |
| **Goldrush** | mid-season | 197 | 560 | 41.33 | 0.90 | -1.97 | 0.56 | -1.11 | -1.95 | 3.57 |
| **Harmony** | mid-season | 2 | 5 | 33.98 | 0.88 | -2.10 | 0.62 | -1.01 | -2.03 | 3.64 |
| **Kanona** | mid-season | 2 | 5 | 20.55 | 0.91 | -1.99 | 0.60 | -1.29 | -1.87 | 3.63 |
| **Kennebec** | mid-season | 68 | 189 | 33.30 | 0.71 | -1.88 | 0.58 | -0.93 | -2.12 | 3.64 |
| **Keuka Gold** | mid-season late | 2 | 5 | 31.26 | 0.88 | -2.08 | 0.33 | -1.26 | -1.56 | 3.69 |
| **Krantz** | mid-season | 2 | 6 | 28.01 | 0.93 | -1.88 | 0.42 | -0.78 | -2.18 | 3.50 |
| **Lamoka** | late | 2 | 5 | 29.10 | 0.84 | -2.26 | 0.32 | -1.14 | -1.60 | 3.84 |
| **Lanorma** | mid-season | 3 | 9 | 36.82 | 1.01 | -2.38 | 0.55 | -1.29 | -1.68 | 3.84 |
| **Mystere** | late | 24 | 68 | 33.64 | 0.63 | -2.01 | 0.51 | -0.80 | -1.78 | 3.43 |
| **Nordonna** | mid-season | 4 | 10 | 31.64 | 0.79 | -2.15 | 0.40 | -0.99 | -1.62 | 3.56 |
| **Norland** | early mid-season | 21 | 48 | 31.10 | 0.84 | -1.94 | -0.10 | -1.05 | -1.60 | 3.86 |
| **Peribonka** | early mid-season | 2 | 6 | 38.81 | 0.95 | -1.87 | 0.49 | -1.13 | -1.98 | 3.54 |
| **Pike** | mid-season | 5 | 14 | 39.98 | 0.95 | -1.95 | 0.58 | -1.43 | -1.89 | 3.71 |
| **Pommerelle** | late | 16 | 44 | 46.83 | 0.60 | -2.22 | 0.62 | -0.86 | -1.51 | 3.35 |
| **Prospect** | mid-season | 9 | 24 | 37.34 | 0.90 | -2.04 | 0.62 | -1.11 | -1.88 | 3.58 |
| **Reba** | mid-season | 9 | 24 | 42.92 | 0.89 | -1.95 | 0.32 | -0.97 | -1.78 | 3.52 |
| **Red Cloud** | mid-season | 4 | 12 | 45.41 | 0.74 | -1.61 | 0.14 | -1.13 | -1.58 | 3.46 |
| **Red Maria** | late | 5 | 15 | 64.59 | 0.85 | -1.77 | 0.53 | -1.28 | -1.97 | 3.65 |
| **Roko** | mid-season | 6 | 18 | 42.51 | 0.67 | -2.30 | 0.81 | -1.02 | -1.55 | 3.37 |
| **Russet Burbank** | late | 26 | 29 | 32.50 | 0.90 | -2.10 | 0.24 | -0.91 | -1.70 | 3.55 |
| **Russet Norkota** | early | 6 | 16 | 49.05 | 0.97 | -1.94 | 0.50 | -1.38 | -1.64 | 3.52 |
| **Shepody** | mid-season | 32 | 112 | 29.50 | 0.72 | -2.12 | 0.59 | -0.97 | -1.81 | 3.58 |
| **Sifra** | mid-season late | 3 | 8 | 50.01 | 0.60 | -2.40 | 0.25 | -0.76 | -1.07 | 3.40 |
| **Snowden** | late | 66 | 188 | 33.26 | 0.68 | -1.93 | 0.49 | -0.90 | -2.06 | 3.67 |
| **Superior** | early mid-season | 199 | 367 | 32.60 | 0.73 | -1.99 | 0.57 | -0.98 | -1.95 | 3.58 |
| **Viking** | mid-season | 2 | 5 | 26.02 | 0.80 | -2.18 | 0.18 | -0.96 | -1.44 | 3.61 |
| **Vivaldi** | early mid-season | 11 | 24 | 59.07 | 0.53 | -2.16 | 0.51 | -0.47 | -1.74 | 3.30 |
| **W 1386** | mid-season late | 4 | 12 | 26.65 | 0.91 | -2.16 | 0.23 | -0.76 | -1.73 | 3.51 |
| **Waneta** | late | 6 | 16 | 31.50 | 0.77 | -2.16 | 0.30 | -0.98 | -1.50 | 3.35 |
| **Yukon Gold** | mid-season | 27 | 78 | 30.66 | 0.68 | -2.17 | 0.44 | -0.89 | -1.61 | 3.47 |
|  |  | 1334 | 3382 |  |  |  |  |  |  |  |
