# Supplementary material for: Cultivar-specific nutritional status of potato (Solanum tuberosum L.) crops
Source: PLoS One. 2020 Mar 13;15(3):e0230458. doi: 10.1371/journal.pone.0230458 (PMC7069643; doi:10.1371/journal.pone.0230458)
Supplement: S3 Table — (DOCX) [file pone.0230458.s003.docx]

**S3 Table: True negatives specimens mean clr values for cultivars**

| **Cultivar*** | **clr_N** | **clr_P** | **clr_K** | **clr_Mg** | **clr_Ca** | **clr_Fv** |
| --- | --- | --- | --- | --- | --- | --- |
| **AC Belmont** | 0.703 | -2.120 | 0.622 | -1.623 | -1.072 | 3.491 |
| **AC Chaleur** | 0.704 | -1.932 | 0.360 | -1.553 | -1.074 | 3.496 |
| **Amandine** | 0.519 | -2.290 | 0.557 | -1.616 | -0.696 | 3.526 |
| **Ambra** | 0.713 | -1.978 | 0.275 | -1.393 | -1.082 | 3.464 |
| **Andover** | 0.904 | -2.203 | 0.743 | -2.194 | -0.850 | 3.601 |
| **Aquilon** | 0.810 | -1.939 | 0.404 | -1.834 | -0.894 | 3.453 |
| **Argos** | 0.819 | -1.754 | 0.402 | -1.698 | -1.131 | 3.362 |
| **Atlantic** | 0.775 | -1.901 | 0.132 | -1.503 | -1.069 | 3.568 |
| **Bijou Rouge** | 0.962 | -2.059 | 0.598 | -2.164 | -1.012 | 3.676 |
| **Carolina** | 0.595 | -2.179 | 0.114 | -1.324 | -0.833 | 3.627 |
| **Chieftain** | 0.790 | -1.870 | 0.385 | -1.879 | -1.037 | 3.611 |
| **Coastal Russet** | 0.693 | -1.838 | 0.413 | -1.844 | -0.973 | 3.550 |
| **Dark Red Chieftain** | 0.953 | -1.748 | 0.307 | -1.920 | -1.351 | 3.759 |
| **Estima** | 0.973 | -2.111 | 0.549 | -1.959 | -1.049 | 3.597 |
| **FL 1207** | 0.768 | -2.060 | 0.252 | -1.502 | -0.920 | 3.461 |
| **FL 1533** | 0.625 | -1.826 | 0.212 | -1.380 | -1.095 | 3.465 |
| **Frontier Russet** | 0.532 | -2.484 | 0.575 | -2.065 | -0.164 | 3.606 |
| **Goldrush** | 0.886 | -1.949 | 0.553 | -1.943 | -1.129 | 3.583 |
| **Harmony** | 0.941 | -2.070 | 0.616 | -2.106 | -1.072 | 3.690 |
| **Kanona** | 0.913 | -1.990 | 0.605 | -1.871 | -1.291 | 3.635 |
| **Kennebec** | 0.654 | -2.051 | 0.693 | -2.041 | -0.873 | 3.617 |
| **Keuka Gold** | 0.916 | -2.061 | 0.297 | -1.591 | -1.199 | 3.637 |
| **Krantz** | 0.930 | -1.879 | 0.415 | -2.178 | -0.784 | 3.496 |
| **Lanorma** | 1.005 | -2.377 | 0.472 | -1.684 | -1.260 | 3.843 |
| **Mystere** | 0.621 | -2.029 | 0.515 | -1.740 | -0.791 | 3.424 |
| **Nordonna** | 0.800 | -2.073 | 0.344 | -1.599 | -0.994 | 3.521 |
| **Norland** | 0.810 | -2.029 | 0.030 | -1.588 | -1.003 | 3.781 |
| **Peribonka** | 0.938 | -1.788 | 0.489 | -1.989 | -1.153 | 3.503 |
| **Pike** | 0.974 | -1.983 | 0.590 | -1.870 | -1.460 | 3.749 |
| **Pommerelle** | 0.590 | -2.231 | 0.588 | -1.487 | -0.830 | 3.369 |
| **Prospect** | 0.910 | -2.011 | 0.530 | -1.908 | -1.113 | 3.591 |
| **Reba** | 0.868 | -1.951 | 0.320 | -1.774 | -0.983 | 3.521 |
| **Red Cloud** | 0.765 | -1.604 | 0.137 | -1.587 | -1.132 | 3.422 |
| **Red Maria** | 0.837 | -1.739 | 0.539 | -1.964 | -1.320 | 3.648 |
| **Roko** | 0.666 | -2.304 | 0.820 | -1.536 | -1.001 | 3.356 |
| **Russet Burbank** | 0.889 | -2.076 | 0.259 | -1.699 | -0.967 | 3.595 |
| **Russet Norkota** | 0.955 | -1.930 | 0.516 | -1.673 | -1.387 | 3.519 |
| **Shepody** | 0.737 | -2.065 | 0.566 | -1.831 | -0.992 | 3.585 |
| **Snowden** | 0.680 | -1.980 | 0.374 | -1.883 | -0.845 | 3.655 |
| **Superior** | 0.733 | -1.968 | 0.572 | -1.891 | -1.038 | 3.593 |
| **Viking** | 0.836 | -2.156 | 0.204 | -1.446 | -1.012 | 3.575 |
| **Vivaldi** | 0.537 | -2.149 | 0.513 | -1.724 | -0.479 | 3.302 |
| **W 1386** | 0.921 | -2.180 | 0.241 | -1.747 | -0.756 | 3.520 |
| **Waneta** | 0.758 | -2.207 | 0.424 | -1.485 | -0.920 | 3.430 |
| **Yukon Gold** | 0.747 | -2.155 | 0.395 | -1.589 | -0.907 | 3.509 |

** Cultivars Lamoka and Sifra had no true negative specimens.*
