# Supplementary material for: Cultivar-specific nutritional status of potato (Solanum tuberosum L.) crops
Source: PLoS One. 2020 Mar 13;15(3):e0230458. doi: 10.1371/journal.pone.0230458 (PMC7069643; doi:10.1371/journal.pone.0230458)
Supplement: Attachment — Submitted filename: Response to Reviewers.docx [file pone.0230458.s004.docx]

Response to reviewers

**Dear editor and reviewer(s),**

We revised the manuscript (PONE-D-19-27444) entitled “Cultivar-specific nutritional status of potato (*Solanum tuberosum* L.) crops” according to the reviewers’ comments. We would like to thank the referees for their careful and constructive reviews.

The responses are detailed with respect to the progression of the manuscript.

Thank you again for your consideration of our revised manuscript.

Best regards,

Serge-Étienne Parent, Eng, PhD

Assistant Professor, Laval University

Faculty of Agriculture and Food Sciences

Department of Soils and Food Engineering

2425, Rue de l’agriculture

Québec, Quebec, G1V 0A6, Canada

**Reply to the evaluation**

**Journal requirements**

1. Please ensure that your manuscript meets PLOS ONE’s style requirements, including those for file naming.

Answer: The files names (specifically of Supplementary Information) and the style requirements have been revised and amended. The Funding, Data availability and Competing interests statements have been deleted from the manuscript and inserted within the cover letter as requested. Sponsors who have websites are detailed. The authors contribution is detailed on the title page instead of making a paragraph below. The short title is deleted from the manuscript. Supporting Information is well detailed after the references section.

2. Funding statement section

a. Please provide an amended statement that declares *all* the funding or sources of support (whether external or internal to your organization) received during this study, as detailed online in our guide for authors at <http://journals.plos.org/plosone/s/submit-now>. Please also include the statement “There was no additional external funding received for this study.” in your updated Funding Statement.
b. Please include your amended Funding Statement within your cover letter. We will change the online submission form on your behalf.

Answer: The amended funding statement is provided within the cover letter.

3. Competing interests section

We note that you received funding from commercial sources: Patate Dolbec Inc., Groupe Gosselin FG, Agriparmentier Inc., Ferme Daniel Bolduc Inc.

a. Please provide an amended Competing Interests Statement that explicitly states this commercial funder, along with any other [relevant declarations](http://www.plosone.org/static/competing.action) relating to employment, consultancy, patents, products in development, marketed products, etc.

Within this Competing Interests Statement, please confirm that this does not alter your adherence to all PLOS ONE policies on sharing data and materials by including the following statement: “This does not alter our adherence to PLOS ONE policies on sharing data and materials.” (as detailed online in our guide for authors <http://journals.plos.org/plosone/s/competing-interests>). If there are restrictions on sharing of data and/or materials, please state these. Please note that we cannot proceed with consideration of your article until this information has been declared.

b. Please include your amended Competing Interests Statement within your cover letter. We will change the online submission form on your behalf.

Answer: The amended Competing Interests Statements are provided within the cover letter.

4. We note that Figure 1 in your submission contains map images which may be copyrighted. We require you to either (a) present written permission from the copyright holder to publish these figures specifically under the CC BY 4.0 license, or (b) remove the figures from your submission

Answer: Figure 1 has been deleted. The description can help locate the study area.

**Additional Editor Comments:**

I was confused somewhat by the description of how trials were selected, and what the yield range was since in the methods, trials that had less than 28 Mg ha^-1^ were dropped from inclusion, yet the low yielding group (high versus low) averaged 24.8 Mg ha^-1^. I assume this means that within the selected trials, there were still many low-yielding cultivars, correct?

Answer: Such a filter was applied in the early stage of the paper, but deactivated later on. Its mention was kept in the paper due to our inattention. We deleted this sentence from the manuscript without any consequence on our results.

5. Review Comments to the Author and responses for more specific observations

**Specific observations**

Line 25-26: “The scarcity of data, in particular for new cultivars, constrains to group cultivars into maturity groups.” Awkward sentence – consider revision.

Answer: The sentence has been deleted from the abstract. The deletion does not affect the understanding.

Line 51: “In particular, foliar gene expression ...” Incomplete sentence – revise grammar.

Answer: The introduction has been totally rewritten with the same arguments to simplify understanding. The sentence (or idea) is implicitly rewritten.

“Compared to other maturity groups, cultivars with longer maturity generally show yield potential that is similar or higher [2-4] because of higher genetic potential [5] related to higher foliar nitrogen status [6] and root acquisition rate [7].”

Line 109: Can the maturity classes be further described by their range of days from planting to maturity?

Answer: We added this information (between brackets) to the revised manuscript.

“Five maturity classes were represented, and we matched the duration from planting to harvest described by the Canadian Food Inspection Agency [1], although the names differed: early season (65 – 70 days), early mid-season (70 – 90 days), mid-season (90 – 110 days), mid-season late (110 – 130) and late season (130 days and more) cultivars.”

Line 113-114: If I understand the protocol correctly, this leaf sampling was taken at different times throughout the growing season due to the differences in maturity of the potato cultivars?

Answer: Exactly! The original dataset has some trials where leaf samples were taken at 10%, 50% (middle), and 100% (full) of the blooming stage, or just before haulm killing. The diagnostic leaf of potato is sampled at the beginning (10%) of the blooming stage regardless of the length of the growing season. So, because of the differences in maturity of cultivars, this stage comes earlier or later. Practitioners are able to identify the appropriate sampling periods. The sentence has been rewritten in the manuscript for better understanding.

“The potato diagnostic tissue is the first mature leaf (4^th^ leaf from top) sampled at the beginning (10%) of the blooming stage.”

Line 115-116: “ …, ground to less than 1 mm” – what does that mean exactly? The plant material was ground to less than 1 mm particle diameter? Just curious.

Answer: Exactly! The plant material was ground to pass through a 1 mm sieve before analysis. The sentence has been amended in the manuscript to improve understanding.

“Twenty to 30 leaves were collected at random in each plot, composited, dried at 65^o^C, ground to pass through a 1 mm sieve, and analyzed for N, P, K, Ca and Mg concentrations after dissolution of combustion.”

Lines 229-231: The yield class prediction with the testing set varied widely among cultivars reaching up to 100%, but these very high-level accuracy cultivars have less than 20 samples. Confusing statement.

Answer: We wanted to explain as stated in the amended format that:

“there was high variation in model fit by cultivar as shown in Fig 2. The accuracy at testing varied from 25% for Estima and Waneta, to 100% for Ambra, Carolina, Dark Red Chieftain, Harmony, Peribonka and Viking. All these cultivars had specifically small sample sizes in the dataset, as shown in the S2 Table.”

Line 265-266: “… information additional to maturity grouping is needed to assess nutrient requirements of potato cultivars.” Can you provide some additional considerations and why? Are they practical?

Answer: Since there are large discrepancies within the fertilizer requirements for cultivars of the same maturity group (described before this sentence) involving genetic factors, we argue that “information additional to maturity grouping is needed to assess nutrient requirements of potato cultivars.” As complementary arguments inserted into the manuscript,

“Huang and Salt [51] reported that ionomics allows the discovery of genes controlling natural variation in the plant ionome and for Salt et al. [9], ionomics could capture information about the functional state of an organism driven by genetic and environmental factors. The content of plant tissue reflects what the plant can absorb from the soil and for each nutrient, there is a correlation between its concentration and yield. Moreover, since plant tissue analysis is also carried out to observe the effect of fertilizer applications, and for adjusting the in-season or next season nutrient requirements [52, 53], ionomes could be useful in discriminating potato cultivars.”

The paragraph is merged with the following by:

“Indeed, using a small data set of eight potato cultivars, Hernandes et al. [11] showed that foliar nutrient profiles varied widely among cultivars of the same maturity group.”

Then, in the next paragraph, we added:

“Based on the assumptions of differential genotypic potential, root development, nutrient requirements, nutrient uptake and use efficiency, the goal was to discover interesting structures in the N, P, K, Mg and Ca contents of the diagnosis tissue in order to decipher dissimilarities between cultivars [33].”

which requires clustering algorithms.

Line 269 (and in other statements): The citation style was rather odd, with a double mention of the author.

Answer: All the references cited in the text have been amended. The problem came from an insufficient bibliography setting with our reference manager software.

Line 278-280: The paragraph starts with describing the variation in cultivars and foliar nutrient profiles and would help if there’s more of a transition in explaining to the reader how this variation could be explained through the clustering process. Sentence 278-280 is a stark transition in thought. Consider the addition of another sentence to guide the reader.

Answer: The section has been amended with an insertion before line 278:

“To cluster is to recognize that objects are sufficiently similar to be put in the same group, and to identify distinctions or separations between groups of objects [57, 58]. Based on the assumptions of differential genotypic potential, root development, nutrient requirements, nutrient uptake and use efficiency, the goal was to discover interesting structures in the N, P, K, Mg and Ca clr variables in order to decipher dissimilarities between cultivars [33].”

It would be clear now reading the full section.

Line 290: Could the different methodology of quantifying P (colorimetry/ICP) have contributed to the insignificance of its content in predicting tuber yield classes?

Answer: This section has been amended, and the answer inserted as discussion:

“The P content of the diagnostic leaf did not appear useful in predicting potato tuber yield classes. Other elements (N, K, Ca and Mg) showed important contribution of their clr values to the prediction quality metric, especially N, which is directly related to photosynthesis [56]. Since the fertilization trials were conducted over a time span of 47 years (1970-2017), the question arises whether the different methodology of quantifying P (colorimetry/ICP) may have contributed to depreciating this variable in predicting tuber yield classes. The ICP method is shown to be faster and to give higher results for total phosphorus content in ‘soil’ extracts in comparison to the colorimetric method. However, there are exceptions and controversial results [60-62]. Ivanov et al. [61] found that the two methods for total P determination in plant material were highly correlated, and the results were generally within 5 to 10% of one another. Moreover, Valkama et al. [63] reported that, although agricultural practices, soil conditions and analytical techniques have undergone substantial changes over time, the differences between old and recent experiments in yield responses to P application were not statistically important. For all these reasons, we consider the two analytical methods equally relevant to the analysis. The low importance of the P clr variable in predicting tuber yield classes may come from its correlation with Ca. Globally, the selection of relevant features is achieved, by first checking the correlation between features and response to select the features that have correlation above a selected level (e.g., 0.5). Then, the independent variables need to be uncorrelated with one another. If some features are correlated, only one is kept. The process selected the clr_Ca variable (alphabetical order) instead of clr_P since these features are correlated, as shown in Fig 1B.”

Lines 300-04: Confused with pre-selection procedure - also ties into the question that Reviewer #1 had for lines 305-307.

Answer: Two sections ‘2.5 Ionome effect and yield prediction’ and ‘2.6 Rebalancing a composition: the enchanting islands’ of Methodology have been amended to deal with the confusion. The main amendment inserted in the ‘2.5 Ionome effect and yield prediction’ section is:

“The four quadrants defined by Swets [36] in binary system diagnosis to delineate the response classes were applied to the context in a contingency table (Table 1). The accuracy is the proportion of correctly-predicted instances:

$Accuracy =\frac{TN+TP}{TN+TP+FN+FP}$	(2)

As in medical sciences, the negative term is used in cases where no intervention is needed after diagnosis.

Table 1: Term definitions used for the study.

|  | | **Observed yield** | |
| --- | --- | --- | --- |
|  |  | **Low (unbalanced)** | **High (balanced)** |
| **Predicted yield** | **Low** | **True positive (TP)**: observed low-yielders correctly predicted as low-yielders. | **False positive (FP)**: observed high-yielders incorrectly predicted as low-yielders. |
|  | **High** | **False negative (FN)**: observed low-yielders incorrectly predicted as high-yielders. | **True negative (TN)**: observed high-yielders correctly predicted as high-yielders. |

”

Reviewer #1: In lines 305-307, the authors state that the sample size is small, and results should be carefully interpreted. It would be misleading to the reader to indicate any relationship or conclusions at this stage until further investigation/research is done to explore such implications from this data. Expand on why the predictive accuracy for some cultivars were very high while others were not. Would these potential factors need to be considered in future yield prediction models?

Answer: The section has been amended with a deeper discussion from this sentence:

“The confusion matrix built for cultivars revealed poor predictive accuracy for certain cultivars (i.e., 25% for Estima and Waneta) and conversely an accuracy of 100% for others (i.e., Ambra, Peribonka) as shown in Fig 2. These cultivars involved mainly small sample sizes (only one, two or three high-yielders and five, six or lightly more low-yielders). The problems of small-data in machine learning are numerous, but mainly revolve around over-fitting. The training and testing datasets division could only aggregate observations of one class in the training set so that the model would train to always predict this dominant class [71]. The model could also memorize labels, which is not ideal for generalizing from new data. Brownlee [72] explained that imbalanced classifications (one or less examples in a minority class for hundreds or more examples in the other) pose a challenge for predictive modeling as most of the machine learning algorithms used for classification were designed around the assumption of an equal number of examples for each class. This results in models that have poor predictive performance, specifically for the minority class. The controversial accuracy level for some cultivars (especially low level) could also come from other yield limiting factors specific to the experiments but not involved in this study, as for false positive specimens. Our model was not effective for these cultivars treated separately.”

We added at the end of the section (next paragraph):

“Other algorithmic, sampling and quality measurement approaches could further be implemented to deal with the problems of small-data and unequal distribution of classes [71, 72]. One could extend the predictors to the experimental conditions (soils, weather data), fitting a site-and-cultivar-specific nutrients diagnosis model.”

Lines 319-323: Seems like a transition is missing to connect the different thoughts.

Answer: A transition has been added for a better connection of arguments:

… “The scientific principle behind tissue analysis is that healthy plants contain predictable concentrations of analytical nutrients [73]. The values are compared to established norms for inadequate, adequate and excess levels. However,” Parent et al. [13] proved that …

Conclusion section

Lines 367-369: Could the perturbation vector of leaf compositional space assist with correcting for in-season nutrient imbalances (per cultivar) to improve yield potential?

This section needs further expansion and discussion. What are the implications of the study on potato fertility and management? What about future directions and next steps? Does this research provide a positive direction in precision potato production in Canada? Does this mean that cultivar-specific nutrition recommendations may need revision or can be more precise in the future? What are the economic implications of this research for the potato industry? Are there any?
Why use potato? Has there been significant background research already conducted in this species or has there been cultivar-specific fertility variability that has led to the investigations with potato? Has there been economic impact of varied fertility regimes on potato cultivars that more precise fertility recommendations based on this model could address? Could the dataset have been more robust if collected from other geographical sites? Would it be possible to do some similar analyses with select potato cultivars grown in very controlled conditions and compare these results also to the analyses here?

Answer: All the others raised for the conclusion section are addressed in the amended conclusion. We have added the following expansion:

“Tissue testing remains an informative diagnostic and preventive tool with real-world applications for growers in evaluating the effectiveness of their nutrient management program. When using the right interpretation, this timely and correct tissue testing helps diagnose the presence and magnitude of suspected nutrient deficiencies. By using the compositional perturbation vector involving interactions among nutrients, our study provided a useful tool in potato precision fertilization in Quebec. The perturbation vector can help identify limiting nutrients requiring correcting measures as a season progresses or for subsequent seasons. Moreover, our study implicitly provided robust multi-nutrient norms for potato crops, gathering more cultivars of different maturity classes than the previous works. These norms are sets of true-negative or nutritionally-balanced compositions per cultivar (enchanting islands) with high-yield potential. More data are needed to fine-tune the models, especially for poorly-documented cultivars. New algorithms, other sampling methods and model quality measures could be tested to deal with the problem of small-data and imbalanced classification. Further studies extending predictive features to site-specific conditions could improve the diagnosis with a site- and cultivar-specific nutrient diagnosis model.”

To enhance the reproducibility of your results, we recommend that if applicable you deposit your laboratory protocols in protocols.io, where a protocol can be assigned its own identifier (DOI) such that it can be cited independently in the future.

Answer: We focus mainly on the computation procedure and think that this procedure is clearly detailed in the [GitHub repository](https://github.com/rgoals/2019_PotatoCrop-nutritional-status) (<https://git.io/Jvt2r>). However, it was good to keep us informed of this possibility. We will use it for all practical purposes when our laboratory protocol presents specific innovations.

Finally, after proofreading, your questions and comments helped us significantly improve the manuscript.
